# Increased Incidence of Rhinovirus Pneumonia in Children During the COVID-19 Pandemic in Mexico

**DOI:** 10.1155/2024/8841838

**Published:** 2024-10-30

**Authors:** Fanny Yasmin Ortega-Vargas, Aldo Agustin Herrera-González, Ilen Adriana Díaz-Torres, Isamu Daniel Cabrera-Takane, Patricia Bautista-Carbajal, Miguel Leonardo García-León, Daniel E. Noyola, María Susana Juárez-Tobías, Verónica Tabla-Orozco, Pedro Antonio Martínez-Arce, María del Carmen Espinosa-Sotero, Gerardo Martínez-Aguilar, Fabian Rojas-Larios, Luis Alfonso Salazar-Soto, Rosa María Wong-Chew

**Affiliations:** ^1^Infectious Diseases Research Laboratory, Research Division, Facultad de Medicina, Universidad Nacional Autónoma de México, Coyoacán, Mexico; ^2^Centro de Investigación en Ciencias de La Salud y Biomedicina, Facultad de Medicina, Universidad Autonoma de San Luis Potosi, San Luis Potosí, Mexico; ^3^Hospital Central Dr. Ignacio Morones Prieto, Pediatrics Department, San Luis Potosí, Mexico; ^4^Hospital Pediátrico de Coyoacán, Emergency Department, Mexico City, Mexico; ^5^Hospital Civil de Guadalajara, Pediatric Infectious Diseases Department, Guadalajara, Jalisco, Mexico; ^6^Hospital General de México Dr. Eduardo Liceaga, Pediatrics Department, Mexico City, Mexico; ^7^Hospital Municipal Del Niño de Durango, Emergency Department, Durango, Mexico; ^8^Facultad de Medicina y Nutrición, Universidad Juárez Del Estado de Durango, Durango, Mexico; ^9^Hospital Regional Universitario de Los Servicios de Salud de Colima, Pediatrics Department, Colima, Mexico

**Keywords:** children, HRV, incidence, Mexico, pandemic, pneumonia, respiratory, virus

## Abstract

**Background:** Human rhinovirus (HRV), traditionally recognized as the primary etiological agent of the common cold, has become the second most important viral agent in bronchopulmonary conditions, such as pneumonia and asthma exacerbations. During the COVID-19 pandemic, several viruses exhibited changes in their epidemiological behavior. This study aims to evaluate the clinical and epidemiological characteristics of children with HRV pneumonia before and during the pandemic in Mexico.

**Methods:** A comparative ambispective longitudinal epidemiological study of two cohorts (prepandemic and pandemic periods) was carried out. Two databases were compared: one from 2010 to 2013 and the other from 2021 to 2023. Children under 5 years of age diagnosed with HRV pneumonia were included. Student's *t*-test, *χ*^2^ tests, and logistic regression were used to assess risk factors associated with severe pneumonia. Incidence density was calculated as HRV cases per 10 new cases of pneumonia per month for each year.

**Results:** During the pandemic, the age of presentation shifted from 5 months to 16 months. There was a higher incidence of HRV pneumonia in children during the pandemic, particularly in the second half of 2021, with a peak in July and August. In addition, there was an increase in severity (53% vs. 63%, *p*=0.006) and coinfections (51.3% vs. 76% *p* < 0.001). A higher prevalence of all risk factors was observed in the second cohort.

**Conclusions:** During the pandemic, a shift toward older age, a higher percentage of coinfections, and increased severity associated to HRV pneumonia were observed. These findings highlight the need for the development and implementation of targeted prevention and treatment measures for HRV.

## 1. Introduction

Pneumonia is a major public health concern due to its high morbidity and mortality rates, both in Mexico and globally. It remains the leading cause of illness and hospitalizations in children under 5 years. A 2015 study by the Centers for Disease Control and Prevention (CDC) in the United States identified human rhinovirus (HRV) as one of the primary etiological agents, accounting for 27% of cases of pneumonia and exacerbations of chronic lung conditions [1]^.^

HRV primarily known as the leading cause of the common cold, is a positive-sense, single-stranded RNA virus without an envelope belonging to the Picornaviridae family, Enterovirus genus [2]. While HRV infections are typically mild and self-limiting [3]; it has been increasingly associated with lower respiratory tract infections such as bronchiolitis, pneumonia, asthma exacerbations, and chronic obstructive pulmonary disease (COPD) [4].

In Mexico, data from the Ministry of Health revealed that between 2010 and 2019, there were 1,485,290 reported cases of pneumonia and bronchopneumonia, with the most affected populations being children under 5 years and adults over 65 years [5]. A study carried out by our group between 2010 and 2013 in 11 hospitals across Mexico identified HRV/enterovirus as the second most common cause of pneumonia in children under 5 years, detected in 16% of cases [6].

HRV infections occur year-round but exhibit seasonal variation, peaking during the fall and winter months [7]. However, the circulation of respiratory viruses shifted significantly during the COVID-19 pandemic. In 2020 and 2021, SARS–CoV-2 dominated respiratory disease activity, while other respiratory viruses experienced reduced or atypical circulation [6, 8]. During the COVID-19 pandemic, the detection of enveloped viruses such as influenza or RSV and nonenveloped viruses including rhinovirus, enterovirus, bocavirus, and adenovirus declined. Following the initial decline in virus detection, HRV levels returned to prepandemic levels or even higher in several countries, with an increase in the average age of affected children and a rise in emergency department visits for asthma/reactive airway disease [9]. In some regions, HRV cocirculated with SARS–CoV-2, becoming the most frequently detected virus in ambulatory pediatric patients (49.5%), followed by SARS–CoV-2 (22%), with 7% of coinfections and 10% of hospitalizations [10]. In pediatric patients in the intensive care unit (PICU), HRV was the most prevalent virus during the pandemic, often found in coinfection with other viruses [11], and even higher incidence in PICU patients than prior to the emergence of SARS–CoV-2 in some reports, a pattern was not seen with other respiratory viruses [12].

These changes in HRV epidemiology may be attributed to the implementation of public health and social mitigation measures, such as mask-wearing, hand hygiene, social distancing, school closures, and isolation, which reduced the circulation of many respiratory viruses. By 2022, the CDC reported an increase in the percentage of positive HRV test results in pediatric sentinel surveillance, surpassing levels seen between 2018 and 2020 [13].

This study aims to compare the clinical and epidemiological characteristics (age of presentation, seasonality, severity, and incidence) of HRV in children under 5 years with pneumonia, focusing on the SARS–CoV-2 pandemic period (2021–2023) versus the prepandemic period (2010–2013) to detect changes in the virus' epidemiology through a comparative analysis across several hospitals in Mexico.

## 2. Methods

### 2.1. Study Design

A comparative ambispective longitudinal epidemiological study was conducted on two cohorts to evaluate the rhinovirus incidence, demographic characteristics, clinical features, and risk factors associated with severe pneumonia before and during the pandemic.

### 2.2. Study Population

Data from two cross-sectional studies were compared. The first cohort included children with pneumonia from March 2010 to August 2013, across 11 hospitals in Mexico (Hospital Central “Dr. Ignacio Morones Prieto” San Luis Potosí, Hospital Regional Universitario de los Servicios de Salud del Estado de Colima, Hospital General de Durango, Nuevo Hospital Civil de Guadalajara “Juan I. Menchaca,” Hospital Pediátrico de Coyoacán, Hospital General de Mexicali, Hospital de Pediatría del CMNO, Instituto Mexicano del Seguro Social (IMSS), Hospital para el Niño de Toluca, Hospital General de México “Dr. Eduardo Liceaga,” Guadalajara, Hospital de la Niñez Oaxaqueña, and Hospital Infantil de Tlaxcala). This cohort has already been published, and its database was used for comparison [6]. A second cohort, from July 2021 to March 2023, was collected in six hospitals in the country (Hospital Central “Dr. Ignacio Morones Prieto” San Luis Potosí, Hospital Civil de Guadalajara “Fray Antonio Alcalde,” Guadalajara, Jalisco; Hospital Pediátrico de Coyoacán, Hospital General de México “Dr. Eduardo Liceaga”, Hospital Municipal del Niño de Durango, Hospital Regional Universitario de los Servicios de Salud del Estado de Colima).

### 2.3. Case Definition, Inclusion and Exclusion Criteria

Children younger than 5 years with a clinical and/or radiological diagnosis of pneumonia were included. Pneumonia cases were defined by the presence of respiratory symptoms such as respiratory distress, cough, tachypnea, cyanosis, with or without fever within the first week of symptom onset, and/or a chest X-ray showing pulmonary infiltrates, classified as macro/micronodular, lobar, interstitial, multiple foci, pleural effusion, and mixed. Exclusion criteria included inadequate respiratory sample or lack of clinical and demographic data. All demographic and clinical characteristics were collected using a format designed specifically for this study across all the participating hospitals.

### 2.4. Ethical Statement

The study was approved by the ethics and research committees of all participating institutions (Facultad de Medicina UNAM FM/DI/105/2020, Hospital Civil de Guadalajara 052/20, Hospital Municipal del Niño de Durango 001/2021, Hospital Central Dr Ignacio Morones Prieto 37-21, Hospital General de México Dr. Eduardo Liceaga DI/22/505/05/42, Hospital Pediatrico de Coyoacan 101-011-025-21, Hospital universitario de los servicios de salud del estado de Colima CEI 2022/1/CR/CL/EPI/166). Parents or guardians of children with pneumonia were invited to have their children participate in the study. Written informed consent was obtained from parents or guardians of all participating children before any procedures were performed.

### 2.5. Sample Collection

The first cohort represents a published study from 2010 to 2013 where the viral etiology of pneumonia in children younger than 5 years was determined. The database from that study was used for comparison [6].

For the second cohort, children who met the inclusion criteria who attended the emergency department or were hospitalized in the participating hospitals their parents were invited to have their children participate in the study. After informed consent was obtained, data and sample collection were carried out. Nasal swabs were collected from patients, placed in viral transport medium, and stored at −70 °C before being sent to the Infectious Diseases Research Laboratory (LIEI), Faculty of Medicine, UNAM, where they were kept at −70°C until further processing.

Prior to 2010, multiplex PCR techniques for detecting respiratory pathogens were unavailable. In 2010, our research group initiated a study using these techniques to identify pneumonia pathogens, with recruitment ending in 2013. Comparison between the pandemic period and the 2010–2013 period was feasible due to the availability of the same data and the participation of six of the same hospitals. The study aimed to compare HRV behavior during the pandemic with the earlier period, as the Mexican Ministry of Health had observed consistent respiratory virus patterns between 2010 and 2019. Thus, the 2010–2013 database was used for comparison.

### 2.6. Multiplex RT-PCR for the Detection of Respiratory Viruses

In the first cohort, the Anyplex RV16 multiplex RT-PCR assay (Seegene) was used for viral detection. In the second cohort, samples were processed using the Allplex Respiratory Full Panel multiplex RT-PCR kit (Seegene) at the Infectious Diseases Research Laboratory, Faculty of Medicine, UNAM. The manufacturer's instructions were followed for viral detection. Seegene's STARMag 96X4 Universal Cartridge kit was used for the automatic RNA extraction, followed by real-time multiplex PCR using 50–60 *μ*L of the sample in 96-well plates on Bio-Rad's C1000 Thermo Cycler. The assay detects 19 respiratory viruses (influenza A, H1, H1N1, H3, and B; parainfluenza 1, 2, 3, and 4; adenovirus; bocavirus; respiratory syncytial virus A and B; metapneumovirus A/B; coronavirus NL63, 229E, and OC43; rhinovirus; and enterovirus), and the Allplex also includes 7 bacteria (*M. pneumoniae, B. pertussis, B. parapertussis, C. pneumoniae, H. influenzae, S. pneumoniae*, and *L. pneumophila*).

The equipment's software automatically interpreted the real-time amplification signals, generating reports for the 26 pathogens and the internal controls (IC).

### 2.7. Statistical Analysis

Univariate and bivariate statistical analyses were conducted using the Statistical Package for Social Sciences (IBM® SPSS® version 25). The *χ*^2^ test or Fisher's exact test was used for comparing categorical variables such as sex, area of hospitalization, clinical characteristics, pneumonia severity, and radiographic pattern, while Student's *t*-test was used to compare continuous variables such as age, height, weight, temperature, and respiratory rate between cohorts. A *p* value < 0.05 was considered statistically significant.

In the first cohort, bacterial pathogens were not detected, and bacteria positive samples were excluded from the second cohort to enable comparison of coinfection rates. A logistic regression analysis was performed to assess risk factors for severe pneumonia. HRV incidence density was calculated as the number of HRV cases per 10 new pneumonia cases per month in both cohorts.

## 3. Results

In the first cohort, which spanned from 2010 to 2013, a total of 1404 children under 5 years of age with a clinical and/or radiological diagnosis of pneumonia were included. HRV was detected in 233 cases (16%), and these were subsequently included in the analysis. In the second cohort, from July 2021 to March 2023, 579 children under 5 years of age with a clinical and/or radiological diagnosis of pneumonia were included. HRV was detected in 350 cases (60.44%), which were also included in the analysis.

### 3.1. Demographic and Clinical Characteristics

In the first cohort (2010–2013), a total of 460 patients were diagnosed with pneumonia and HRV detection, either as a single infection or a coinfection, compared to 350 patients in the second cohort (2021–2023). A higher proportion of girls was observed in the second cohort, with 159 (34.5%) versus 148 (42.29%) girls and 301 (65.4%) versus 202 (57.71%) boys (*p*=0.025). In addition, there was a shift toward older age in the second cohort, with the mean age ± standard deviation (SD) increasing from 5.75 ± 11.84 to 16.93 ± 16.11 months (*p* < 0.001). Height and weight also increased in the second cohort, with mean heights of 0.69 ± 0.14 versus 0.75 ± 0.18 m (*p* < 0.001) and weight of 7.86 ± 3.44 versus 9.43 ± 5.74 kg (*p* < 0.001), respectively.

Most patients in both cohorts were hospitalized: 438 patients (95.22%) versus 335 (95.71%) were admitted, while four patients (0.87%) versus four patients (1.14%) were treated as outpatients, and 18 (3.91%) versus 11 (3.14%) patients were admitted to the intensive care unit in the first and second cohorts, respectively (Table 1).

Body temperature (mean ± SE) was 37.32 ± 0.93°C in the first cohort and 36.85 ± 0.78°C in the first and second cohort. Differences between the two periods were noted in clinical characteristics: the respiratory rate was 49.93 ± 13.15 versus 40.94 ± 14.72 breaths per minute, cough was present in 438 (95.22%) versus 318 (91.12%), thoracoabdominal dissociation in 223 (48.48%) versus 140 (40.35%), intercostal retraction in 335 (72.38%) versus 219/62.75%), xiphoid retraction in 140 (30.43%) versus 138 (39.77), nasal flaring in 230 (50%) versus 118 (33.91%) patients, and dysphonia in 59 (12.83%) versus 48 (13.83) patients in the first versus the second cohort, respectively. Severe pneumonia cases increased from 53.48% (246/460) before the pandemic to 63.14% (221/350) during the pandemic, according to the World Health Organization (WHO) criteria [14]. In addition, coinfections increased from 236 (51.3%) patients in the first cohort to 266 (76%) in the second cohort. The radiographic analysis showed an interstitial pattern in 163 (35.4%) versus 164 (46.85%), a lobar pattern in 42 (9.13%) versus 50 (14.28%), multiple foci in 108 (23.48%) versus four cases (1.14%), micro- and macronodular patterns in 62 (13.48%) versus 25 cases (7.14%), and mixed patterns in 42 (9.12%) versus 39 cases (11.14%) in the first and second cohorts, respectively (Table 2).

### 3.2. Risk Factors

Several risk factors associated with severe pneumonia were higher in the first cohort (2010–2013) compared to the second cohort (2021–2023). Immunocompromised status was present in 8.5% of cases (OR 1.08, 95% CI 0.55–2.10) versus 3.6% of cases (OR 1.16, 95% CI 0.34–3.95), absence of breastfeeding in 46.3% (OR 1.50, 95% CI 1.03–2.19) versus 24.8% (OR 1.59, 95% CI 0.91–2.76), biomass use in 17% (OR 0.49, 95% CI 0.31–0.76) versus 12% of cases (OR 1.10, 95% CI 0.56–2.16), absence of influenza vaccine in 74.3% (OR 1.21, 95% CI 0.80–1.82) versus 63.3% of cases (OR 1.09, 95% CI 0.6–1.757), and comorbidities in 34% (OR 1.38, 95% CI 0.92–2.05) versus 20% (OR 0.83, 95% CI 0.49–1.39) of cases, respectively. However, some risk factors were lower in the first cohort compared to the second cohort, including domestic smoking in 29.2% (OR 1.16, 95% CI 0.77–1.76) versus 41.6% of cases (OR 1.43, 95% CI 0.91–2.26), daycare attendance in 4.8% (OR 0.71, 95% CI 0.30–1.68) versus 9.5% of cases (OR 0.96, 95% CI 0.46–1.99), incomplete vaccination schedule in 37.8% (OR 0.96, 95% CI 0.65–1.40) versus 51.1% of cases (OR 1.81, 95% CI 1.15–2.82), respectively. Although the 95% confidence intervals overlap, indicating no significant differences between the two periods (Table 3).

### 3.3. Seasonality

**Figure 1:**
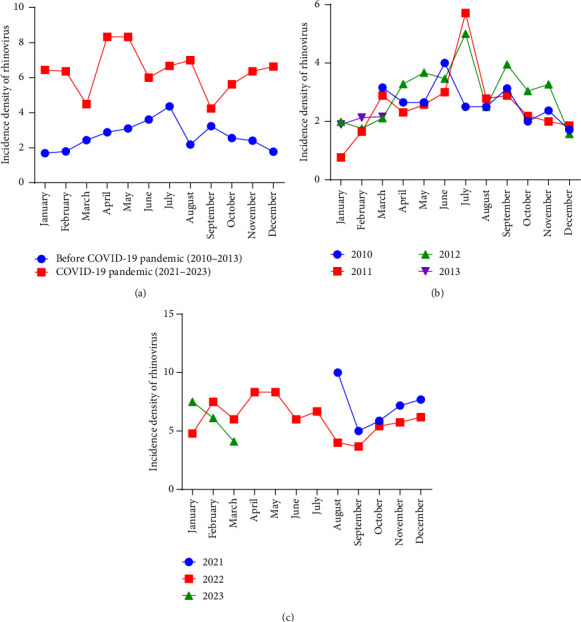
Incidence density of rhinovirus infection in children with pneumonia before and during the COVID-19 pandemic. (a) Comparison of the mean incidence of HRV infection in children with pneumonia in the prepandemic period (2010–2013) and the pandemic period (2021–2023); (b) incidence of HRV in children with pneumonia before the pandemic during the 2010–2013 period; (c) incidence of HRV in children with pneumonia during the pandemic in the 2021–2023 period. The incidence density was calculated as the HRV cases per 10 new pneumonia cases per month for each year within the study periods. This comparative analysis provides insights into the trends and shifts in HRV infection rates in pediatric pneumonia cases before and during the COVID-19 pandemic.

During the prepandemic period (2010–2013), HRV was detected throughout the entire year, with a peak in the summer of each year. In 2021, there was a marked increase in HRV incidence (10 versus six cases per 10 new pneumonia cases per month) during July and August, followed by a second peak from October to December 2021, corresponding to the relaxation of social distancing, measures imposed during the COVID-19 pandemic. This trend of continuous HRV incidence persisted throughout 2022 and into early 2023. Peaks were observed from October to January in both the 2021 and 2022 seasons, compared to the 2010–2013 period, with an overall increase in case incidence during the pandemic compared to previous years (Figure 1).

## 4. Discussion

This study presents a comparative analysis of the clinical and epidemiological characteristics of HRV infections in children under five years with pneumonia, focusing on the prepandemic period (2010–2013) and the pandemic period (2021–2023). Utilizing data from a secondary database and a prospective cohort initiated in 2021, the study aims to explore the impact of the pandemic on HRV infections.

Between 2021 and 2023, HRV was identified in 60.44% of children under 5 years of age diagnosed with pneumonia, marking a significant increase of 16% during the pandemic period. This surge in HRV cases during the pandemic aligns with findings from studies conducted in China, Spain, and Australia, which also reported an upward trend in HRV incidence in children, particularly in children under 10 years, contrasting with the decline of other respiratory viruses. The persistence of HRV during the pandemic has been attributed to its structural properties, which confer greater resistance compared to enveloped viruses [1, 15, 16]. Nonenveloped viruses such as HRV are generally more virulent, are shed from infected individuals for a longer time, can survive in the gastrointestinal tract, and are more resistant to extreme conditions such as pH, heat, dryness, and disinfectants. All these factors favor the persistence of these viruses on surfaces and extended shedding among infected subjects, even amid nonpharmacological interventions [9].

A notable demographic shift was observed, with the average age of HRV presentation increasing from 5 months in the prepandemic period to 16 months during the pandemic. This age shift may be attributed to the so called “immunological debt” created by the implementation of social distancing and sanitation measures during the COVID-19 pandemic, which limited children's exposure to the pathogens. This phenomenon has been documented by other researchers, including Lamrani Hanchi et al. who reported a decrease in respiratory virus infections under six months during the pandemic, followed by an increase in cases among 1 to 2 years olds [14].

Regarding disease severity, the study found an increase in severe pneumonia cases during the pandemic (63.14%) compared to the prepandemic period (53.48%) based on WHO criteria [17]. This rise in severity may be partially explained by the higher rate of coinfections observed during the pandemic (76% versus 51.3%), as coinfections have been associated with greater disease severity [18]. In addition, the lack of immunity due to reduced pathogen exposure during the pandemic may have contributed to the higher severity of HRV infections.

According to the World Health Organization, there are risk factors that increase children's susceptibility to pneumonia, including the use of biomass, overcrowding, and exposure to tobacco, among others [19]. Other authors also consider prematurity, low birth weight, lack of breastfeeding, and family history [20]. In this study, an increase in risk factors, including absence of breastfeeding (59.4% versus 71.4%), use of biomass (40% versus 65.1%), and incomplete vaccination schedule (52.5% versus 70.2%) were identified as risk factors associated with severity.

Seasonal trends in HRV incidence also shifted during the pandemic. While HRV circulated year-round in the prepandemic period, the pandemic period saw a significant decrease in cases during the spring–summer months, followed by an increase in the fall. In the prepandemic period, HRV incidence fluctuated but remained consistent throughout the years. A peak in HRV cases was observed in August–September 2021, coinciding with the lifting of nonpharmacological interventions, including the full reopening of schools in June 2021 [21].

In conclusion, regarding the impact of the COVID-19 pandemic on rhinovirus infections, the implementation of nonpharmacological measures such as confinement, face masks, and social distancing disrupted herd immunity in the pediatric population, contributing to more severe outcomes secondary to a previously unexposed immune system and the presence of coinfections. Furthermore, there was a shift in the age of presentation, which is directly related to the duration of confinement and the lack of exposure to the environment, so when schools reopened, there was a corresponding increase in incidence. Continued surveillance of respiratory viruses, particularly HRV, is crucial for the development of prevention and treatment strategies.

A key limitation of this study is that it did not include all the pneumonia patients but those diagnosed with a clinical and/or radiological pneumonia whose parents agreed consented to participation. This limitation affects the calculation of HRV incidence, as the total number of children at risk during the study period was not available. Instead, incidence density was calculated using the new monthly pneumonia cases as the at-risk–population, irrespective of the etiology. In addition, the number of patients from each participating center was limited, restricting the ability to analyze regional distribution. Despite these limitations, the study serves as a valuable multicenter reference for understanding the impact of the SARS–CoV-2 pandemic on HRV infections in Mexico.

## 5. Conclusions

HRV has emerged as a major etiological agent in pediatric respiratory diseases, particularly severe pneumonia. During the COVID-19 pandemic, a shift toward older age groups, an increase in coinfections, and greater disease severity associated with HRV pneumonia were observed. Ongoing surveillance and research are essential to inform the development of preventive and therapeutic strategies against HRV. This study underscores the burden of HRV in children and highlights the need for further investigations into preventive measures and treatment options.

## Figures and Tables

**Table 1:** Demographic characteristics of children admitted with HRV-associated pneumonia: Comparison between two cohorts before and during the COVID-19 pandemic.

Characteristics	2010–2013 (*n* = 460)	2021–2023 (*n* = 350)	*p*
*Sex, n (%)*			
Female *n* (%)	159 (34.5)	148 (42.29)	**0.025**⁣^∗^
Male *n* (%)	301 (65.4)	202 (57.71)	
Height (meters, *x* ± SD)	0.69 ± 0.14	0.75 ± 0.18	**< 0.001**⁣^∗^
Weight (kilograms, *x* ± SD)	7.86 ± 3.44	9.43 ± 5.74	**< 0.001**⁣^∗^
Age (months, *x* ± SD)	5.75 ± 11.84	16.93 ± 16.11	**< 0.001**⁣^∗^

*Hospital area*			
Ambulatory *n* (%)	4 (0.87)	4 (1.14)	0.785
Hospitalization *n* (%)	438 (95.22)	335 (95.71)	
Intensive care unit *n* (%)	18 (3.91)	11 (3.14)	

*Note:* This table presents the demographic characteristics of two cohorts of children under five years of age diagnosed with pneumonia caused by human rhinovirus (HRV) before (2010–2013) and during (2021–2023) the COVID-19 pandemic. The table compares variables such as gender distribution, age, height, weight, and hospitalization status across the two cohorts.

Statistically significant differences are noted where ⁣^∗^*p* value < 0.05.

**Table 2:** Clinical characteristics of children with HRV-associated pneumonia: Comparison between two cohorts before (2010–2013) and during the COVID-19 pandemic (2021–2023).

Clinical characteristics	2010–2013 (*n* = 460)	2021–2023 (*n* = 350)	*p*
Temperature (°C, *x* ± SD)	37.32 ± 0.93	36.85 ± 0.78	0.562
Respiratory rate (bpm, *x* ± SD)	49.93 ± 13.15	40.94 ± 14.72	**< 0.001**
Cough, *n* (%)	438 (95.22)	318 (91.12)	**0.02**
Thoracoabdominal dissociation, *n* (%)	223 (48.48)	140 (40.35)	**0.021**
Intercostal retraction, *n* (%)	335 (72.83)	219 (62.75)	**0.002**
Xiphoid retraction, *n* (%)	140 (30.43)	138 (39.77)	**0.006**
Nasal flaring, *n* (%)	230 (50)	118 (33.91)	**< 0.001**
Dysphonia, *n* (%)	59 (12.83)	48 (13.83)	**< 0.001**
Coinfections *n* (%)	236 (51.3)	266 (76)	**< 0.001**

*WHO severity score*			
Severe *n* (%)	246 (53.48)	221 (63.14)	**0.006**
Nonsevere *n* (%)	214 (46.52)	129 (36.86)	

*Radiographic pattern*			
Micro- and/or macronodular *n* (%)	62 (13.48)	25 (7.14)	**< 0.001**
Multiple foci *n* (%)	108 (23.48)	4 (1.143)	
Lobar *n* (%)	42 (9.13)	50 (14.286)	
Interstitial *n* (%)	163 (35.4)	164 (46.857)	
Mixed *n* (%)	42 (9.13)	39 (11.143)	
NA *n* (%)	43 (9.35)	67 (19.14)	

*Note:* This table outlines the clinical characteristics of children diagnosed with pneumonia caused by human rhinovirus (HRV) before and during the COVID-19 pandemic. Key clinical features such as body temperature, respiratory rate, and the presence of symptoms such as cough, thoracoabdominal dissociation, and retractions, as well as radiological patterns, are compared between the two cohorts. The bold values are the statistically significant differences.

A *p* value < 0.05 is considered statistically significant. NA indicates cases where chest X-rays were not obtained.

**Table 3:** Risk factors associated with severe pneumonia.

Risk factors	2010–2013			2021–2023		
	S (%)	NS (%)	OR (95% CI)	S (%)	NS (%)	OR (95% CI)
	*n* = 246	*n* = 214		*n* = 221	*n* = 129	
	*n* (%)	*n* (%)		*n* (%)	*n* (%)	
Immunocompromised	21 (8.5)	17 (7.9)	1.082 (0.555–2.108)	8 (3.6)	4 (3.1)	1.166 (0.344–3.952)
Breastfeeding absence	114 (46.3)	78 (36.4)	**1.506 (1.035–2.190)**	55 (24.8)	22 (17)	1.591 (0.916–2.762)
Domestic smoking	72 (29.2)	56 (26.1)	1.167 (0.775–1.760)	92 (41.6)	43 (33.3)	1.438 (0.913–2.263)
Biomass use	42 (17)	63 (29.4)	**0.493 (0.317–0.769)**	28 (12.6)	15 (11.6)	1.108 (0.568–2.163)
Daycare attendance	12 (4.8)	12 (5.6)	0.713 (0.302–1.686)	21 (9.5)	13 (10.0)	0.961 (0.463–1.993)
Incomplete vaccination schedule	93 (37.8)	84 (39.2)	0.963 (0.659–1.407)	113 (51.1)	48 (37.2)	**1.811 (1.159–2.829)**
Influenza vaccine absence	183 (74.3)	151 (70.5)	1.212 (0.804–1.827)	140 (63.3)	81 (62.7)	1.090 (0.677–1.757)
Comorbidities	86 (34.9)	60 (28.0)	1.380 (0.927–2.052)	46 (20.8)	31 (24.0)	0.831 (0.495–1.395)

*Note:* This table presents the risk factors associated with severe pneumonia in children with human rhinovirus (HRV) infections. The odds ratio (OR) and 95% confidence interval (CI) were calculated to estimate the probability of developing severe pneumonia for each risk factor. The bold values are those which are significant because the 95% CI do not cross the number 1.

Abbreviations: NS, nonsevere cases; S, severe cases. A higher OR suggests a greater likelihood of developing severe pneumonia in the presence of the respective risk factor.

## Data Availability

The data that support the findings of this study are available from the corresponding author upon reasonable request.

## References

[1] Takashita E., Kawakami C., Momoki T (2021). Increased Risk of Rhinovirus Infection in Children During the Coronavirus Disease-19 Pandemic. *Influenza Other Respiratory Viruses*.

[2] Hayashi Y., Sada M., Shirai T (2022). Rhinovirus Infection and Virus-Induced Asthma. *Viruses*.

[3] Ljubin-Sternak S., Meštrović T. (2023). Rhinovirus-A True Respiratory Threat or a Common Inconvenience of Childhood?. *Viruses*.

[4] Bizot E., Bousquet A., Charpié M (2021). Rhinovirus: A Narrative Review on Its Genetic Characteristics, Pediatric Clinical Presentations, and Pathogenesis. *Frontiers in Pediatrics*.

[5] Ministy of Health (2021). Specific Action Program. Prevention and Control of Acute Respiratory Infections (Pneumonia, Influenza and COVID-19) 2020-2024. https://www.gob.mx/cms/uploads/attachment/file/706929/PAE_IRA_cF.pdf.

[6] Wong-Chew R. M., Garcia-Leon M. L., Noyola D. E. (2017). Respiratory Viruses Detected in Mexican Children Younger Than 5 Years Old With Community-Acquired Pneumonia: A National Multicenter Study. *International Journal of Infectious Diseases*.

[7] Leotte J., Trombetta H., Faggion H. Z (2017). Impact and Seasonality of Human Rhinovirus Infection in Hospitalized Patients for Two Consecutive Years. *Jornal de Pediatria*.

[8] Centers for Disease Control and Prevention (2022). Increased Respiratory Virus Activity, Especially Among Children, Early in the 2022-2023 Fall and Winter. https://emergency.cdc.gov/han/2022/han00479.asp.

[9] Principi N., Autore G., Ramundo G., Esposito S. (2023). Epidemiology of Respiratory Infections During the COVID-19 Pandemic. *Viruses*.

[10] Hammes Varela F., Sauthier Srthor I., Polese-Bonatto M. (2022). Rhinovirus as the Main Co-Criculating Virus During the COVID-19 Pandemic in Children. *Journal De Pediatria*.

[11] Regina Malveste Ito C., Santos M. O., de Oliveira Cunha M. (2024). Rhinovirus Infection and Co-Infection in Children With Severe Acute Respiratory Infection During the COVID-19 Pandemic Period. *Virulence*.

[12] Gil E., Roy S., Best T., Hatcher J., Breuer J. (2023). Increasing Rhinovirus Prevalence in Paediatric Intensive Care Patients Since the SARS-CoV2 Pandemic. *Journal of Clinical Virology*.

[13] Ma K. C., Winn A., Moline H. L (2022). Increase in Acute Respiratory Illnesses Among Children and Adolescents Associated With Rhinoviruses and Enteroviruses. *Morbidity and Mortality Weekly Report*.

[14] Lamrani Hanchi A., Guennouni M., Ben Houmich T. (2022). Changes in the Epidemiology of Respiratory Pathogens in Children During the COVID-19 Pandemic. *Pathogens*.

[15] Brañas P., Muñoz-Gallego I., Espartosa E., Moral N., Abellán G., Folgueira L. (2023). Dynamics of Respiratory Viruses Other Than SARS-CoV-2 During the COVID-19 Pandemic in Madrid, Spain. *Influenza and Other Respiratory Viruses*.

[16] Sullivan S. G., Carlson S., Cheng A. C (2020). Where Has All the Influenza Gone? The Impact of COVID-19 on the Circulation of Influenza and Other Respiratory Viruses. *Euro Surveillance*.

[17] World Health Organization/The United Nation Children’s Fund (2008). *Chart Booklet: Integrated Management of Childhood Illness (IMCI)*.

[18] Arnold F. W., Fuqua J. L. (2020). Viral Respiratory Infections: A Cause of Community-Acquired Pneumonia or a Predisposing Factor. *Current Opinion in Pulmonary Medicine*.

[19] Organización Mundial de la Salud (2022). Neumonia Infantil. https://www.who.int/es/news-room/fact-sheets/detail/pneumonia.

[20] Chen L., Miao C., Chen Y. (2021). Age-Specific Risk Factors of Severe Pneumonia Among Pediatric Patients Hospitalized With Community-Acquired Pneumonia. *Italian Journal of Pediatrics*.

[21] Ministry of Health (2021). Responsible and Ordered Return to School’s Guide. School Year. https://coronavirus.gob.mx/wp-content/uploads/2021/08/GuiaAperturaEscolar-SEP-20agosto202119hrs.pdf.

